# Developmental Expression of 4-Repeat-Tau Induces Neuronal Aneuploidy in *Drosophila* Tauopathy Models

**DOI:** 10.1038/srep40764

**Published:** 2017-01-23

**Authors:** Nicolas Malmanche, Pierre Dourlen, Marc Gistelinck, Florie Demiautte, Nichole Link, Cloé Dupont, Lies Vanden Broeck, Elisabeth Werkmeister, Philippe Amouyel, Antonino Bongiovanni, Hélène Bauderlique, Dieder Moechars, Anne Royou, Hugo J. Bellen, Frank Lafont, Patrick Callaerts, Jean-Charles Lambert, Bart Dermaut

**Affiliations:** 1 Inserm UMR1167, Laboratoire d’Excellence Distalz, Lille, F59000, France; 2 Institut Pasteur de Lille, Longevity Research Center, Lille, F59000, France; 3 Université de Lille, Lille, F59000, France; 4 Laboratory of Behavioral and Developmental Genetics, Center for Human Genetics, KU Leuven, Leuven, 3000, Belgium; 5 VIB Center for the Biology of Disease, Leuven, 3000, Belgium; 6 Department of Molecular and Human Genetics, Baylor College of Medicine, Houston, TX 77030, USA; 7 Center for Infection and Immunity of Lille, 59019, France; 8 Inserm UMR1019, Lille, 59019, France; 9 CNRS UMR8204, Lille, 59019, France; 10 Neuroscience Department, Janssen Research and Development, a Division of Janssen Pharmaceutica NV, Beerse, 2340, Belgium; 11 CNRS UMR5095, Pessac, 33607, France; 12 Institut Européen de Chimie et Biologie, Pessac, 33607, France; 13 Institut de Biochimie et Génétique Cellulaires, Université de Bordeaux, Pessac, 33607, France; 14 Howard Hughes Medical Institute, Houston, TX 77030, USA; 15 Program in Developmental Biology, Baylor College of Medicine, Houston, TX 77030, USA; 16 Department of Neuroscience, Baylor College of Medicine, Houston, TX 77030, USA; 17 Jan and Dan Duncan Neurological Research Institute, Houston, TX 77030, USA; 18 Center for Medical Genetics, Ghent University Hospital, Ghent University, Gent, 9000, Belgium

## Abstract

Tau-mediated neurodegeneration in Alzheimer’s disease and tauopathies is generally assumed to start in a normally developed brain. However, several lines of evidence suggest that impaired Tau isoform expression during development could affect mitosis and ploidy in post-mitotic differentiated tissue. Interestingly, the relative expression levels of Tau isoforms containing either 3 (3R-Tau) or 4 repeats (4R-Tau) play an important role both during brain development and neurodegeneration. Here, we used genetic and cellular tools to study the link between 3R and 4R-Tau isoform expression, mitotic progression in neuronal progenitors and post-mitotic neuronal survival. Our results illustrated that the severity of Tau-induced adult phenotypes depends on 4R-Tau isoform expression during development. As recently described, we observed a mitotic delay in 4R-Tau expressing cells of larval eye discs and brains. Live imaging revealed that the spindle undergoes a cycle of collapse and recovery before proceeding to anaphase. Furthermore, we found a high level of aneuploidy in post-mitotic differentiated tissue. Finally, we showed that overexpression of wild type and mutant 4R-Tau isoform in neuroblastoma SH-SY5Y cell lines is sufficient to induce monopolar spindles. Taken together, our results suggested that neurodegeneration could be in part linked to neuronal aneuploidy caused by 4R-Tau expression during brain development.

Genetic and neuropathological studies have implicated the neuronal microtubule-associated protein Tau (*MAPT*) as a central player in a wide range of adult-onset neurodegenerative disorders including Alzheimer’s disease (AD), frontotemporal dementia (FTD) and related tauopathies such as progressive supranuclear palsy (PSP) and corticobasal degeneration (CBD)^1^. In post-mitotic neuronal cells, Tau is involved in microtubule stability and its microtubule associated function is regulated by phosphorylation and differential isoform expression^2^. In addition to its predicted MAP activity, Tau is increasingly acknowledged for its multi-functional protein activities^3^. Among those functions, there is medical and biological evidence linking Tau to genomic stability. First, Tau has been reported to have a DNA-protective function in the nucleus^4^,^5^. Second, it has been shown that aneuploidy is present in peripheral cells and in primary fibroblast cell cultures from patients carrying known Tauopathies mutations^6^,^7^. Third, in mice, expression of similar Tau mutations also induces genomic instability ranging from chromosome rearrangements to aneuploidy^8^. Finally, a recent report showed that human Tau overexpression in *Drosophila* can induce a mitotic block leading to aneuploidy^9^.

Isoform expression is developmentally regulated with the 3R-Tau being expressed in the developing fetal brain while equal amounts of 3R- and 4R-Tau isoforms are present in post-mitotic differentiated neurons^2^. It has been shown that some autosomal dominant forms of FTD are caused by heterozygous *MAPT* mutations that affect the alternative splicing of exon 10, favoring increased levels of 4R-Tau. Moreover, a common inversion polymorphism at 17q21.31 encompassing the Tau gene gives rise to two common haplotypes, called H1 and H2, with H1 being associated with increased risk of the 4R-Tauopathies PSP and CBD^10^,^11^. Functional analysis has shown that within the H1 haplotype, *MAPT* exon 10 inclusion is favored leading to increased 4R-Tau expression^12^.

In *Drosophila,* studies of tauopathy models based on panneuronal expression of human 4R-Tau with or without clinical FTD-causing mutations, have shown induction of post-mitotic cell cycle re-entry, mitochondrial toxicity, chromatin relaxation and lamin dysfunction^13^,^14^,^15^,^16^,^17^. In general, these proposed pathways are assumed to take place in a normally developed and genomically stable postnatal or adult brain. However, in these panneuronal *Drosophila* tauopathy models, induction of transgene expression relies on the binary Gal4/UAS system with Tau expression being induced by the Gal4 transcription factor that is under the control of the panneuronal Elav promoter. While it has been reported that the panneuronal Elav protein is only detected in the nuclei of post-mitotic neurons^18^, it was shown that the panneuronal *Elav-Gal4* enhancer trap driver induces Gal4 expression and activity prior to post-mitotic neuronal differentiation^19^,^20^. Therefore, the phenotypes described for 4R-Tau expression using the *Elav-Gal4* driver could also involve a potential role for 4R-Tau isoform during neuronal proliferation or developmental post-mitotic differentiation. Interestingly, using the same system, a recent study has shown cellular ablation of adult neuronal structures called mushroom bodies arising during embryonic development suggesting an important role for 4R-Tau during neurodevelopment^21^. In addition, using a gene switch system to control Tau expression, it was shown that 4R-Tau adult-onset expression does not affect adult life span^22^,^23^. Thus, while a strong focus has been put on deciphering 4R-Tau toxicity in post-mitotic neuronal cells, it remains unclear whether adult 4R-Tau toxicity requires a neurodevelopmental component.

In the present study, we report that 4R-Tau-mediated adult phenotypes in established *Drosophila* tauopathy models are pre-determined during larval development where 4R-Tau expression affects normal mitotic progression inducing severe spindle morphology and chromosome segregation defects. Furthermore, neuroblastoma cell lines allowing conditional expression of 4R wild type and clinical Tau mutations causing early-onset FTD, induced identical mitotic spindle defects, suggesting that our fly results could be relevant in a human disease context.

## Results

### Adult photoreceptor loss and reduced life span require 4R-Tau expression during development

**Figure 1:**
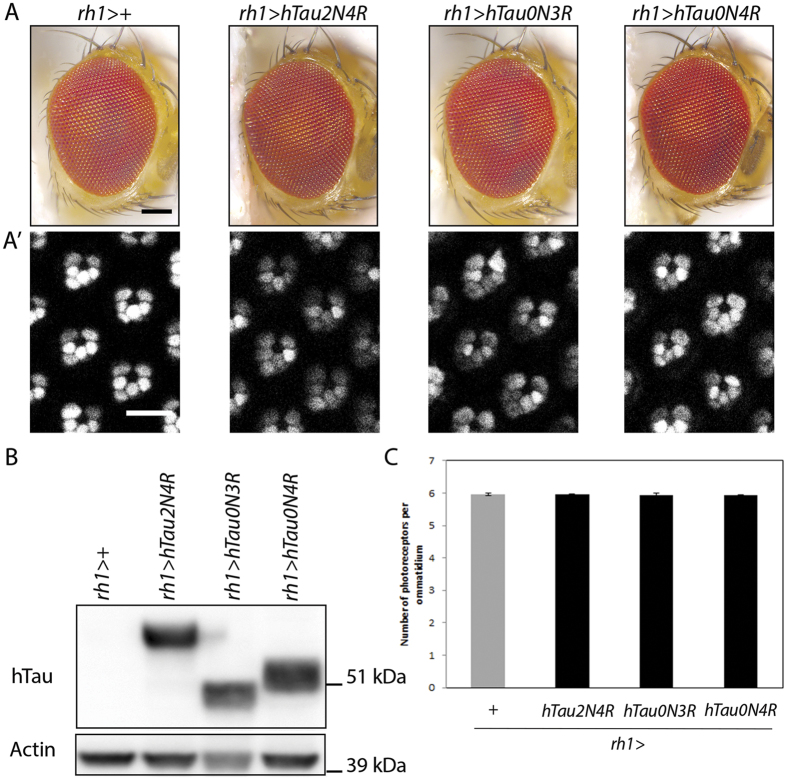
Late pupal-onset expression of 3R or 4R-Tau isoforms does not affect *Drosophila* the external eye phenotype nor photoreceptor survival. (**A**) *Drosophila* external eye phenotype upon expression of 3R or 4R-Tau in differentiated photoreceptors using the *rh1-Gal4* driver (Scale bar = 100 μm). (A’) Adult photoreceptor neurons in living flies imaged using a *rh1*-*GFP* transgene at 28–30 days after continuous late pupal-onset induction of 3R or 4R-Tau expression using the *rh1-Gal4* driver (Scale bar = 10 μm). (**B**) Western blot from adult fly heads showing the presence of Tau protein isoforms upon induction of transgene expression using the *rh1-Gal4* driver. The corresponding full-length western blot is presented in Sup Fig. 5A. (**C**) Quantification of photoreceptors at 28–30 days showing no loss of photoreceptor neurons upon late pupal-onset induction of 3R or 4R-Tau expression (n = 8 fly eyes, mean ± SD).

We first tried to develop a novel model to screen for adult-onset Tau-mediated neurodegeneration in *Drosophila* using a sensitive cornea neutralization method to quantify progressive photoreceptor loss in living flies^24^. For this, we induced expression of human wild-type 3R or 4R-Tau isoforms using the binary UAS/Gal4 system^25^ in differentiated photoreceptors using the late pupal *rh1-Gal4* driver (*rh1>Tau*). In this genetic background, control and *rh1>Tau* adult eyes appear normal in their external organization (Fig. 1A), a situation allowing us to quantify potential photoreceptor loss in 28 to 30 days old flies (Fig. 1A’). Interestingly, our results show that driving expression of either 3R or 4R Tau isoforms in differentiated photoreceptors did not lead to photoreceptor loss despite high levels of Tau protein isoforms (Fig. 1B,C).

**Figure 2:**
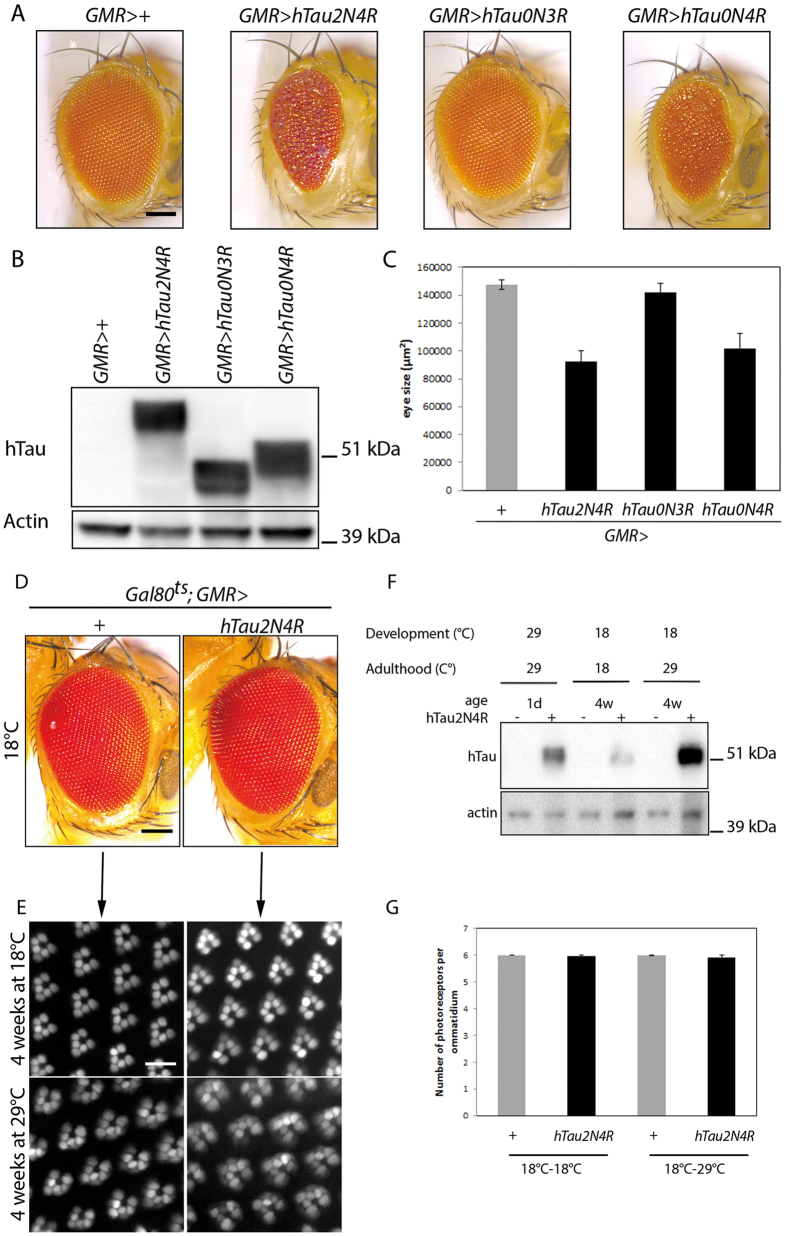
Only 4R-Tau expression during development impact external adult eye phenotype. (**A**) *Drosophila* external eye phenotype upon 3R or 4R-Tau expression in retinas during development using the *GMR-Gal4* driver (Scale bar = 100 μm). (**B**) Western blot from adult fly heads showing the presence of Tau protein isoforms upon induction of transgene expression using the *GMR-Gal4* driver. (**C**) Quantification of eye size upon 3R or 4R-Tau expression using the *GMR-Gal4* driver (n = 10 fly eyes, mean ± SD). (**D**) *Drosophila* external eye phenotype upon inhibition (18 °C) of 4R-Tau expression in retinas during development (Scale bar = 100 μm). (**E**) Adult photoreceptors in living flies imaged using a *rh1*-GFP transgene at 4 weeks after continuous inhibition (18 °C) or adult-onset induction of 4R-Tau expression (29 °C) (Scale bar = 10 μm). (**F**) Western blot from adult heads showing the level of 4R-Tau isoform after induction in the three genotypes studied. The corresponding full-length western blot is presented in Sup Fig. 5A. (H) Quantification of photoreceptor neurons at 4 weeks showing no loss of photoreceptor neurons upon adult-onset induction of 4R-Tau expression (n = 6 fly eyes, mean ± SD).

To investigate a requirement for Tau expression during development, we induced 3R or 4R-Tau expression using the *GMR-Gal4* eye specific driver. As previously described, driving 4R-Tau isoform during development induces an adult rough eye phenotype (Fig. 2A), associated with vacuolar degeneration in the underlying lamina as previously described^26^,^27^. Furthermore, 4R-Tau expression is associated with a reduction of eye size (Fig. 2C) while 3R-Tau isoform does not affect eye development despite a high level of 3R-Tau protein upon induction (Fig. 2B). To confirm this result, we used the temperature-sensitive TARGET system for spatio-temporal control of transgene expression^28^ to investigate the developmental versus adult-onset 4R-Tau phenotype in two models: (1) eye-specific expression using the *GMR-Gal4* driver (*GMR>Tau*) and (2) panneuronal expression using the *Elav-Gal4* driver (*Elav>Tau*). *GMR>Tau* flies in which 4R-Tau expression was repressed during development had normal external eyes, as expected (Fig. 2D). We took advantage of this normal eye phenotype to induce 4R-Tau expression during adulthood to quantify photoreceptor loss over a period of 4 weeks by cornea neutralization. Once again, the number of photoreceptors did not decrease despite high levels of Tau protein over the experimental time period (Fig. 2E–G).

**Figure 3:**
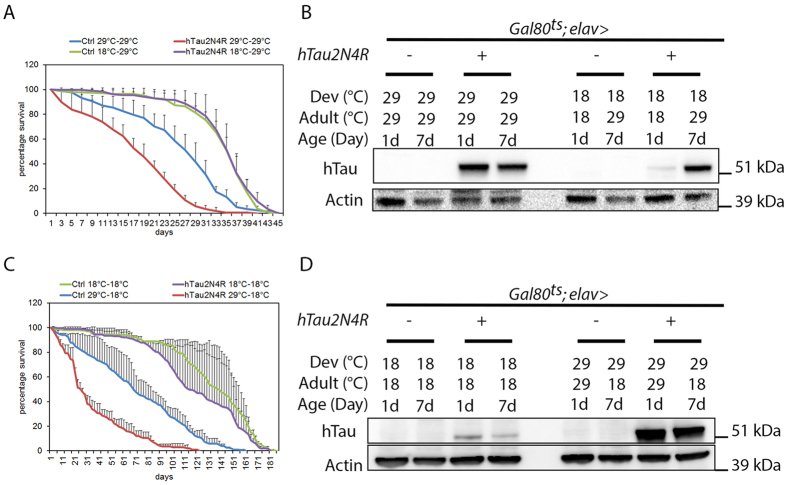
4R-Tau expression is required during development to impact adult lifespan. (**A**) Life span experiment (n = 300 adult females, mean ± SD) showing that 4R-Tau expression throughout development and adulthood (29 °C-29 °C) induced a reduction in the mean life span while adult-onset expression of 4R-Tau (18 °C–29 °C) does not affect the mean life span when compared to control flies. (**B**) Western blot from adult fly heads showing 4R-Tau adult expression upon temperature switch. The corresponding full-length western blot is presented in Sup Fig. 5B. (**C**) Life span experiment (n = 200 adult females, mean ± SD) showing that developmental 4R-Tau expression (29 °C–18 °C) is sufficient to induce a reduction in the mean life span. (**C**) Western blot from adult fly heads showing the persistence of 4R-Tau proteins in adult head despite inhibition of expression upon temperature switch. The corresponding full-length western blot is presented in Sup Fig. 5B.

It has been shown that panneuronal (*Elav>Tau*) Tau expression throughout all stages of the *Drosophila* life cycle induces a reduced adult lifespan, mushroom body defects and vacuolar neurodegeneration in adult brains^21^,^29^,^30^. Using the TARGET system, our results suggest that the previously reported reduction in adult lifespan^29^ depends, to a large extent, on 4R-Tau expression during development. Indeed, panneuronal expression of 4R-Tau throughout development and during adulthood significantly reduced lifespan in contrast to panneuronal adult-onset expression, which had no negative impact on lifespan (Fig. 3A,B) as reported previously^22^,^23^,^31^. Furthermore, in the reciprocal experiment, we observed that 4R-Tau expression during development is sufficient to impact adult life span since shutting down expression in adulthood did not improve adult life span expectancy (Fig. 3C). However, in this context, we could still detect a similar level of Tau proteins at birth and after four weeks of transgene repression (Fig. 3D and data not shown), a result preventing us to conclude that 4R-Tau expression during development is the only event inducing a reduced lifespan. Finally, we also noted a decreased lifespan in control flies when raised and aged at 29 °C (Fig. 3A). This confirms that both temperature and Gal4 protein can have adverse effects as reported previously^32^,^33^. Nonetheless, our results using both experimental settings strongly suggested that developmental expression of 4R-Tau is required and appears sufficient to induce photoreceptor loss and reduced lifespan in *Drosophila*.

### 4R-Tau delays mitotic progression by affecting spindle dynamics in *Drosophila* neuronal progenitors

**Figure 4:**
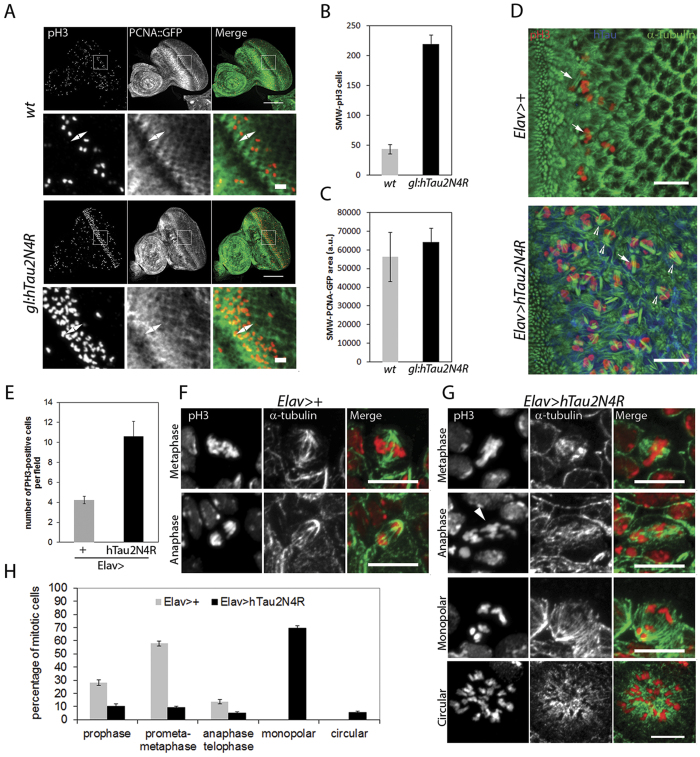
4R-Tau induced mitotic phenotypes in larval eye imaginal discs and brains. (**A**) Larval eye discs from wild-type (n = 8) and 4R-Tau (n = 9) flies were analyzed for S phase (using a *PCNA*-EmGFP transgene)^66^ and M phase (using pH3 staining) (Scale bar = 50 μm). The double arrow indicates the SMW. (**B**) Quantification of pH3-positive mitotic cells within the SMW in wild-type and 4R-Tau expressing eye discs (mean ± SD). (**C**) Quantification of S phase cells within the SMW in wild-type and 4R-Tau expressing eye discs (mean ± SD). (**D**) Spindle morphology in wild-type mitotic cells reveals the presence of normal bipolar spindles (arrows) while in 4R-Tau expressing eye discs, a large portion of the mitotic cells display a monopolar spindle configuration (arrowheads) (Scale bar = 10 μm). (**E**) Mitotic index in wild-type and 4R-Tau expressing third instar larval brains (n = 8 for each genotypes, mean ± SD) indicates an accumulation of pH3-positive cells. (**F**,**G**) Images of mitotic figures in wild-type and 4R-Tau-expressing larval brains. (**F**) In wild-type, metaphase cells show the presence of a well-focused bipolar spindle and at anaphase the segregation of two equal DNA masses at opposite poles. (**G**) In 4R-Tau expressing ganglion mother cells, the metaphase spindle has only one well-focused pole and at anaphase the two DNA masses appear to be unequal and asynchronous in their movement towards opposite poles (arrowhead). Furthermore, most cells display a monopolar configuration. Circular mitotic figures showing an increased DNA content are also observed (Scale bar = 10 μm). (**H**) Quantification of the mitotic phases in wild type (n = 1171, mean ± SEM) and 4R-Tau (n = 1985, mean ± SEM) expressing larval brains reveals a high frequency of dividing cells with a monopolar spindle configuration.

It has been recently reported that 4R-Tau over expression in *Drosophila* wing discs induces a mitotic delay characterized by the formation of monopolar spindles leading to aneuploidy and cell death in a non-neuronal cell context^9^. In addition, the authors observed a similar spindle phenotype within the larval neuronal progenitors but without further investigation. Thus, we first asked if these mitotic phenotypes are induced independently of Tau isoforms or if they are specific to 3R or 4R-Tau as observed in our eye phenotype and photoreceptor ageing experiments. For this, we first used third instar larval eye imaginal discs as a model to quantify M cell cycle phases during the second mitotic wave (SMW). The SMW is the last round of cell division localized posterior to the morphogenetic furrow, prior to cell differentiation and ommatidia organization. We detected M phase using a phospho-histone3 Ser10 (pH3) antibody labeling mitotic chromosomes in wild-type, 3R and 4R-Tau genetic background using both the eye specific and the panneuronal drivers. Our results showed a strong increase of pH3 positive mitotic cells only for 4R-Tau isoform expression (Sup Fig. 1A–D). To further distinguish between a mitotic delay and an overproliferation phenotype, we quantified both S and M cell cycle phases in *gl-Tau* eye discs. For this, we detected S phase using a GFP-tagged proliferating cell nuclear antigen (PCNA) and M phase as above. Our results show a strong increase in pH3 positive mitotic cells using these transgenic flies, while the level of S phase cells remained normal (Fig. 4A–C). Also, we observed the presence of monopolar spindles and hypercondensed chromosomes upon 4R-Tau expression similar to the ones reported in a non-neuronal cell context (Fig. 4D)^9^. These results indicate that 4R-Tau expression induces an increase of mitotic cells in the developing retina, likely due to delayed mitosis rather than an increased cell proliferation.

To explore the mitotic phenotype associated with 4R-Tau expression in more detail, we studied third instar larval brains by driving 4R-Tau expression using the panneuronal *Elav-Gal4* driver. First, we analyzed the *Elav-Gal4* driver activity. As previously reported, the *Elav-Gal4* driver induced Gal4 activity prior to neuronal differentiation as observed by the accumulation of a membrane GFP tag reporter transgene for Gal4 activity in neuroblasts^20^ (Sup Fig. 2). Furthermore, this Gal4 activity is sufficient to drive 4R-Tau expression within neuroblasts and ganglion mother cells (Sup Fig. 2). In line with our results driving 3R or 4R-Tau isoforms in eye specific context or panneuronal, we found that *Elav>4R-Tau* larval brains showed an increased number of mitotic cells per optical field in contrast to 3R-Tau larval brains and that the vast majority of mitotic cells displayed hypercondensed chromosomes (Fig. 4E and Sup Fig. 3A,B). We next examined and quantified mitotic spindle configurations and mitotic phases in wild type and *Elav>4R-Tau* larval brains. Our results clearly showed the presence of two abnormal mitotic configurations upon 4R-Tau expression: monopolar spindles and circular mitotic figures (Fig. 4F–H). Furthermore, at metaphase we rarely observed a normal bipolar spindle but rather spindles with only one well-focused pole (Fig. 4G), and finally, we observed that at anaphase, chromosome segregation was asynchronous (Fig. 4G).

**Figure 5:**
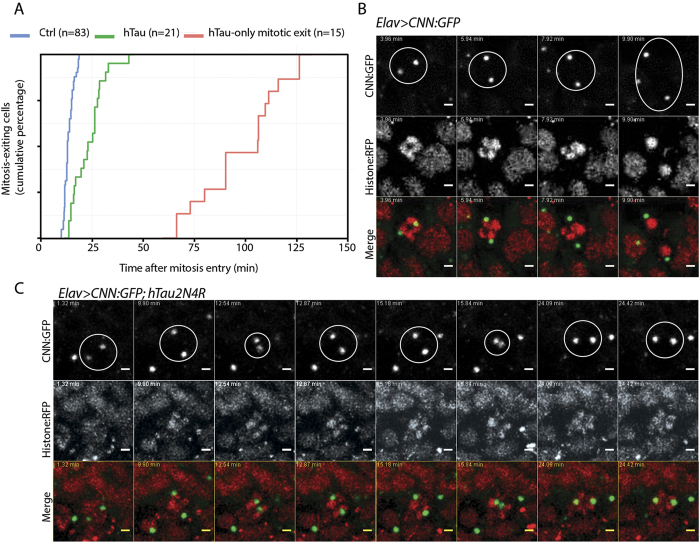
4R-Tau expression delayed mitosis and centrosome dynamics. (**A**) Cumulative mitotic timing was calculated in real time using a Jupiter:GFP and Histone:RFP transgene. The mitotic timing started when two Jupiter:GFP foci were on each side of the DNA and ended at telophase. (**B**) Centrosome movement during mitosis in control cells showing centrosome separation to opposite poles. Each centrosome remains at its pole following nuclear envelop breakdown. (**C**) Centrosome movement during mitosis in 4R-Tau expressing cells showing that centrosomes are going through several cycles of collapse and separation prior to anaphase onset.

To gain a better understanding of the mitotic progression upon 4R-Tau expression, we analysed mitosis in real-time in larval brains using tagged tubulin and histone transgenes allowing us to time mitotic progression from prophase until telophase. During mitosis, the microtubule network is re-organized to establish a bipolar spindle such that each kinetochore pair faces opposite spindle poles, a configuration satisfying the spindle assembly checkpoint requirement to trigger the metaphase-anaphase transition. In wild-type animals, neuronal progenitors go through mitosis within 15 to 17 minutes (Fig. 5A and Sup Movie S1). However, in *Elav>4R-Tau* cells, we identified four cell populations: (1) cells for which we only observed mitotic exit with asynchronous chromosome movement after 1 to 2 hours of prometaphase/metaphase duration (Fig. 5A, Sup Movie S2), (2) cells with an almost normal mitotic timing (15 to 20 minutes) and a normal anaphase progression (Sup Movie S3 arrowhead), (3) cells with a mitotic timing ranging from more than 20 minutes up to 35 minutes and for which chromosome hypercondensation and spindle collapse are visible at metaphase Sup Movie S2 arrow), (4) and finally a cell population displaying circular mitosis (Sup Movie S4).

To further characterize the event associated with the spindle collapse and recovery, we analysed centrosome dynamics in real-time using a centrosomin tag transgene^34^. As previously described, following duplication, centrosomes separate towards opposite poles before nuclear envelope breakdown and spindle formation (Fig. 5B, Sup Movie S5). Interestingly, in 4R-Tau expressing cells, we observed a separation of the centrosomes toward opposite poles prior to nuclear envelope breakdown as in wild type condition. This situation appears similar to the microtubule dynamics we described above. However, at metaphase, the centrosomes moved location and underwent a cycle of separation/collapse prior to anaphase onset (Fig. 5C, Sup Movie S6). Taken together, our results in neuronal progenitors of larval eye discs and brains demonstrated that 4R-Tau expression delayed mitotic progression by inducing spindle and centrosome collapse at metaphase as well as chromosome hypercondensation.

### 4R-Tau expression results in an increased level of aneuploidy in developing and post-mitotic neuronal tissue

**Figure 6:**
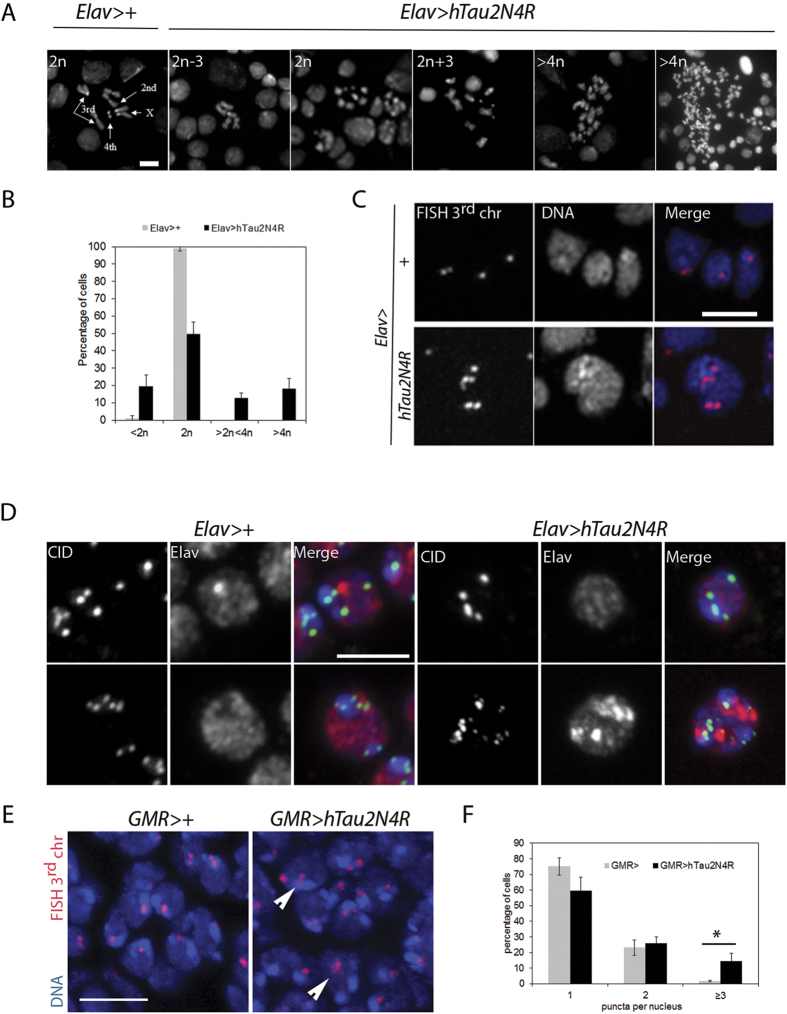
Aneuploidy generated by 4R-Tau expression in larval brains and post-mitotic pupal retinas. (**A**) Metaphase spread from wild-type third instar larval brains showing the karyotype composed of 4 well identifiable chromosome pairs. Examples of diploid and aneuploid karyotypes following 4R-Tau expression during *Drosophila* development (Scale bar = 10 μm). (**B**) Quantification of the aneuploidy in wild type control (n = 206, mean ± SD) and 4R-Tau-expressing brains (n = 302, mean ± SD). (**C**) FISH experiment to detect the third chromosome Dodeca satellite in adult brains in wild-type and 4R-Tau adult flies. An aneuploid adult neuron in 4R-Tau adult brains with 5 third chromosomes is shown (Scale bar = 10 μm). (**D**) Detection of the CID/Cenp-A epitope to visualize all centromeres indicated the presence of different centromeric configurations in wild-type adult neurons. Example of adult neurons in brains with developmental 4R-Tau expression exhibiting a wild-type configuration (upper panels) and an aneuploid neuron showing the presence of 15 clear CID/Cenp-A foci (lower panels) (Scale bar = 5 μm). (**E**) Wild type and 4R-Tau-expressing pupal retinas were assayed for chromosome abnormalities using FISH to detect the third chromosome Dodeca satellite probe labeled with Cy3. Arrowheads indicate aneuploid cells. Scale bar = 5 μm. (**F**) Quantification of FISH foci in wild type and 4R-Tau expressing pupal retinas. 4R-Tau expressing animals contained significantly more nuclei with 3 or more foci, indicating an increase in aneuploidy. Unpaired two-tailed t-test, p = 0.0113.

Next, we asked if the mitotic phenotypes induced by 4R-Tau expression could lead to chromosome segregation errors in larval brains. Ploidy in *Elav>4R-Tau* larval brains was quantified after hypotonic treatment to allow spreading of metaphase chromosomes. In wild-type larval brains less than 1% of the metaphase spreads showed chromosome loss while 55% of the 4R-Tau metaphase spreads displayed aneuploidy ranging from loss of a single chromosome to a severe chromosomal gain (Fig. 6A,B). These results indicated that 4R-Tau expression resulted in a failure to properly segregate chromosomes to the two daughter cells.

Despite the presence of aneuploidy in the developing larval *Elav>4R-Tau* brains, these animals are able to develop into viable adult flies albeit with a reduced lifespan (Fig. 3A and C). Thus, we looked for aneuploid neurons in adult *Elav>4R-Tau* brains by fluorescent *in-situ* hybridization (FISH) using a 3^rd^ chromosome dodeca satellite probe^35^. Due to somatic pairing in *Drosophila,* wild-type euploid nuclei typically display 1 and sometimes 2 foci (Fig. 6C). As a result FISH does not allow detection of monosomy, while polysomy can be easily scored. Interestingly, 4R-Tau-expressing adult brains contained nuclei with 3 or more foci indicating the presence of aneuploid neurons (Fig. 6C). We could confirm this result by detecting the centromeric protein Cenp-A/CID (Fig. 6D). In addition, we quantified the level of aneuploidy by FISH in post-mitotic cells of wild-type and *GMR>3R or 4R-Tau* pupal retinas (Fig. 6E,F ans Sup Fig. 4). We observed significantly more aneuploid nuclei (~10%) in 4R-Tau-expressing pupal retinas. Taken together, these results demonstrate that 4R-Tau isoform expression leads to an increased number of aneuploid post-mitotic neurons.

### Wild type and FTD mutant Tau isoforms induce monopolar spindles in neuroblastoma SH-SY5Y cell line

**Figure 7:**
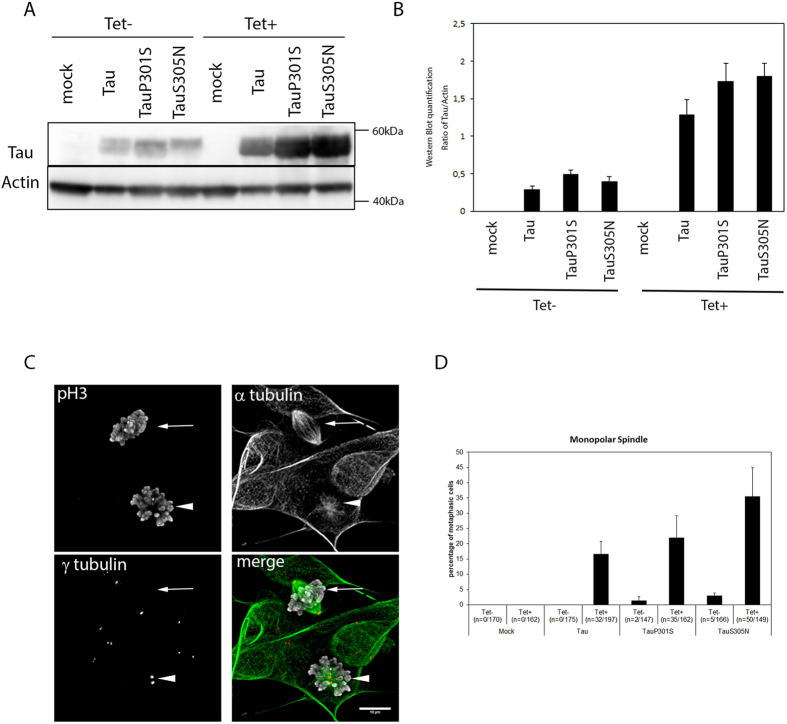
Monopolar spindle phenotype in human SH-SY5Y neuroblastoma cell line model following expression of wild-type and mutant 4R-Tau isoforms. (**A**) Western blot in mock and Tau cell lines showing the increase in Tau protein levels upon tetracycline induction. (**B**) Quantification of Tau protein level upon tetracycline induction. The corresponding full-length western blot is presented in Sup Fig. 5C. (**C**) Representative image of a monopolar spindle (arrowhead) close to a normal bipolar spindle (arrow) in Tau-expressing cells (scale bar = 10 μm). (**D**) Quantification of the percentage of monopolar spindle in the Tau-expressing cells (mean ± SD, the number of monopolar spindles out of the total number of mitotic cells after nuclear envelop breakdown over 3 independent experiments is indicated in the legend of the x-axis).

To extend our fly findings to a human cellular model, we used a Tau-inducible neuroblastoma cell line (SH-SY5Y) allowing expression of wild-type and clinical mutants (p.P301S and p.S305N) of 4R-Tau during cell proliferation^36^. In this system the expression of human 4R-Tau protein was switched on by adding tetracycline to the culture medium (Fig. 7A,B) and the cell culture was analyzed for spindle configuration and frequency 96 h after 4R-Tau induction. To identify mitotic cells, we detected by indirect immuno-fluorescence α-tubulin and γ-tubulin as well as pH3 epitopes. As for our *Drosophila* model, overexpression of wild-type 4R-Tau induced the formation of monopolar spindles with 2 centrosomes (Fig. 7C,D). Interestingly, we also observed monopolar spindles with both clinical 4R-Tau mutations p.P301S and p.S305N (Fig. 7D). Noticeably, the frequency of monopolar spindles was increased compared to wild-type 4R-Tau. However, we could not conclude for a direct effect of the clinical mutation over the observed increase as the level of 4R-Tau mutant proteins was slightly higher than wild type 4R-Tau (Fig. 7B). Nonetheless, these results suggest that increased levels of 4R-Tau during proliferation of neuroblastoma cells could generate aneuploid daughter cells.

## Discussion

Here, we have used published *Drosophila* tauopathy models, based on overexpression of human 3R or 4R-Tau isoforms, to explore the cellular mechanisms of Tau-mediated neurotoxicity. Previous fly studies have allowed the identification of key post-mitotic neurodegenerative processes upon 4R-Tau overexpression. However, these studies assumed that the observed post-mitotic phenotypes take place in an otherwise normal brain^4^,^15^,^16^,^17^,^37^. Interestingly, several studies have challenged that notion. First, it has been shown that 4R-Tau expression during development impairs the correct organisation of the mushroom body, a structure being defined during embryonic development^21^. Second, 4R-Tau over-expression in the epithelia of *Drosophila* imaginal discs during cell proliferation clearly illustrates the induction of a mitotic block and the presence of aneuploidy leading to apoptosis^9^. To compare the impact of developmental versus adult-onset Tau expression in the induction of the visible *Drosophila* adult phenotypes, we used a combination of the binary and the TARGET transgene expression systems to have a more precise spatio-temporal control of Tau isoforms expression during the *Drosophila* life cycle. In our experimental set-up, we found that the induction of Tau phenotypes depends on two keys parameters: first, it is Tau isoform specific and second, it depends on the timing of expression. Indeed, our results show that the 3R-Tau isoform gives rise to normal adult flies independently of timing of expression while 4R-Tau isoform expression strongly affects adult fly phenotypes only when 4R-Tau expression occurs during development. Thus, our results let us conclude that the fly tauopathy model requires 4R-Tau expression during development to exhibit a rough eye, adult photoreceptor loss and reduced life span. However, we also show that 4R-Tau protein level remains constant over time after transgene repression. While this event precludes us to exclude any 4R-Tau toxicity in the processes of the adult Tau phenotypes, our results illustrate that 4R-Tau expression is required and essential during development to trigger a robust adult phenotype in terms of life span reduction and photoreceptor loss. Furthermore, a combination of cytogenetic and live imaging experiments clearly illustrate that 4R-Tau expression affects cell division by inducing a mitotic delay during neurodevelopment in larval eye discs and brains. Interestingly, Tau is known to be hyperphosphorylated during mitosis^38^,^39^,^40^ and to localize to the mitotic spindle^41^. In addition, overexpression of Tau induces ectopic meiotic spindles in a *Xenopus* oocyte maturation assay^42^, reduces adult neurogenesis in a mouse model of tauopathy^43^ and induces a mitotic block in a *Drosophila* model of tauopathy^9^. In this report, we further characterized the mitotic phenotype induced by 4R-Tau expression during development. In particular, we observed that 4R-Tau induced the formation of monopolar spindles at high frequency. This resulted in chromosome missegregation and increased levels of aneuploid neurons in post-mitotic neuronal tissue. In addition, the monopolar mitotic spindle phenotype was observed in the neuroblastoma cell line SH-SY5Y cells upon induction of wild-type and clinically mutant Tau.

Our results on live material indicate that upon 4R-Tau expression, mitosis appears to progress normally until metaphase alignment. At this point, we observed the induction of chromosome hypercondensation and spindle collapses into a monopolar configuration. It appears that the majority of cells recovers from this configuration and moves forward to anaphase onset. The remaining cells stay in a monopolar mitotic configuration for an extended period of time before undergoing anaphase. Monopolar spindles can be caused by defects in centrosome duplication, mitotic kinases, motor-dependent forces or microtubule dynamics^44^. In line with recent evidence of an interaction between 4R-Tau and the motor kinesin Klp69F during mitosis^9^, we hypothesize that 4R-Tau overexpression affects spindle dynamics at metaphase by altering microtubule flux and resulting in the subsequent collapse of the bipolar spindle. Regardless of the underlying mechanism, the 4R-Tau-induced spindle phenotype was associated with a high frequency of aneuploidies (>50%) in larval 4R-Tau-expressing brains. Surprisingly, the resulting adult flies are viable and display only “mild” adult-onset phenotypes including a reduced lifespan and, as reported previously, vacuolar brain degeneration^29^. Although we were able to detect increased levels of aneuploidy in post-mitotic neuronal tissue, more work is needed to address whether neuronal aneuploidy plays a direct causal role in the neurodegeneration seen in these fly models.

Our conclusions contrast with previous studies suggesting that tauopathies are diseases occurring and developing in post-mitotic differentiated neurons. Accordingly, Khurana *et al*. have demonstrated that the cell cycle is activated in post-mitotic neurons of adult 4R-Tau-transgenic fly brains and triggers neuronal apoptosis^15^. In contrast, we observe cell cycle defects in mitotic cells during neurodevelopment when 4R-Tau delays rather than induces cell cycle progression. These profound differences are all the more intriguing given that the *Drosophila* models (drivers and transgenes) used by us and Khurana *et al*. are essentially identical. While it is possible that these phenomena are completely independent, we favor the idea that both aneuploidy generated during neurodevelopment and cell cycle activation in post-mitotic neurons are causally linked. Aneuploidy is a feature of most solid tumors and it has been suggested that the resulting imbalances in oncogenes and/or tumor suppressor genes may promote inappropriate cell proliferation^45^. Possibly, 4R-Tau-induced aneuploidy triggers a similar cell-cycle mechanism in post-mitotic neurons leading to their death.

Although the idea of a neurodevelopmental origin seems counterintuitive for an adult-onset neurodegenerative disorder, recent advances in single-cell sequencing^46^ have revealed that clonal somatic genome variation constitutes a surprisingly widespread feature of the human body including the brain^47^,^48^,^49^. It has been shown that up to ~40% of the neurons of a normal brain harbors some kind of genomic imbalance ranging from copy number variations to gains and losses of chromosomal segments and even entire chromosomes^48^. Furthermore, cell death-prone aneuploid neurons have been documented in the neocortex of AD patients and their frequency appears to correlate with regional vulnerability^50^,^51^,^52^,^53^,^54^ (reviewed in Arendt, 2012). In addition, several lines of evidence support a neurodevelopmental component in AD pathogenesis. Neuroanatomical studies in AD brains indicate that Tau containing neurofibrillary tangles are clustered along the vertical columns^55^,^56^,^57^ that constitute the ontogenetic and clonally related units of the human neocortex^58^. Accordingly, cortical atrophy in AD seems to result from a decrease in cortical length rather than thickness reflecting a progressive loss of these cortical columns^59^. In addition, PSEN1 and APP have been localized to centrosomes^60^,^61^ and (over)expression of Aβ, APP or PSEN1 induced aneuploidy^52^,^53^. Taken together, we hypothesize that tauopathies could be, at least partially, developmental cell cycle diseases of the brain characterized by chromosomal instability.

## Materials and Methods

### Live imaging of fluorescently labelled photoreceptors

CO_2_-anesthetized flies were placed in a 35 mm cell culture dish half-filled with 1% agarose and covered with water at 4 °C according to the cornea neutralization technique^62^. Flies were observed using an upright epi-fluorescent microscope (AxioImager Z1; Zeiss) equipped with a 40× water immersion long-distance objective. Images were acquired using a digital camera (AxioCam MRm, Zeiss) and acquisition software (Zeiss Zen software). Photoreceptor neurons were visually quantified.

### *Drosophila* genetics

*gl-Tau* was described previously and induces eye specific overexpression of full length human 2N4R wildtype *Tau*^26^. The *UAS-2N4RTau* and *gl-Tau* constructs were a kind gift of George R. Jackson. The UAS-0N3RTau construct were a kind gift of A. Mudher^63^. The Jupiter GFP, Histone RFP, UAS-CNN GFP and the Gal4 driver rh1, Elav and GMR, the thermo sensitive Gal 80 transgene were obtained from Bloomington *Drosophila* Stock Center. For the ageing experiment, parental crosses between *Elav-Gal4; Gal80*^*ts*^ virgin females and *w*^*1118*^ or *w*^*1118*^*; UAS-2N4RTau* males were done at 18 or 29 °C. In F1, females were collected every morning, split in groups of 10 per tube before crossing with *w*^*1118*^ males and aged at 18 or 29 °C. Tubes were flipped every two days over the course of the experiment. All flies were raised using the Molasse Formula from Nutri-Fly (catalog number 66–114) supplement with 0.01% Tegosept and 4.8 ml of Propionic acid per liter of food.

### Cytology, histology and FISH analysis

Eye discs from 3^rd^ instar larvae were dissected in Sang Medium and fixed 4% paraformaldehyde for 30 min. Eye discs were washed 3 × 10 min in PBST (PBS + 0.1% Triton). Mouse anti-phospho Histone H3 Ser 10 (Cell signaling, 1:100) and Rabbit anti-Tau (Dako, 1:400) were incubated over-night at 4 °C. Fluorescent secondary antibody (Invitrogen) were used at 1:1000 and incubated for 2 h at RT in PBST. Mouse anti-α tubulin-FITC conjugated (Sigma, clone DM1A) was used at 1:50 and incubated for 1 h at room temperature. Finally, the slides are washed in PBST, and mounted in Vectashield (Vector Laboratories, Burlingame, CA). Images were acquired using a Zeiss 710 confocal microscope. PCNA and PH3 were quantified using Image J software (NIH).

Brains from 3^rd^ instar larvae were dissected in physiological saline solution (0.7% NaCl). Following fixation in 4% paraformaldehyde for 30 min, brains were transferred to 45% acetic acid for 3 min, and briefly immersed in 60% acetic acid. Thereafter, brains were squashed between a coverslip and microscope slide, the slide frozen in liquid nitrogen and following removal of the coverslip incubated in ethanol for 10 min at −20 °C. The brains were rehydrated and permeabilized in PBST for 10 min. Next the slides were washed in PBS and blocked with 1% BSA in PBS for 45 min. Mouse anti-α tubulin (Sigma, clone DM1A) and Rabbit anti-phospho Histone H3 Ser 10 (Millipore) were used at 1:1000 in PBS and incubated for 1 h at RT. The slides were washed 3 × 5 min in PBS. Fluorescent secondary antibody (Invitrogen) were used at 1:1000 and incubated for 1 h at RT in PBS-1% BSA. Finally, the slides are washed in PBS, and mounted in DAPI Vectashield (Vector Laboratories, Burlingame, CA). Images were acquired using a Zeiss 710 confocal microscope.

For hypotonic treatment, brains from 3^rd^ instar larvae were dissected in physiological saline solution (0.7% NaCl) and incubated for 10 min in 0.5% sodium citrate prior to fixation in a mix of acetic acid: methanol: water (11: 11: 2) for 30 seconds. Then, the slide was frozen in liquid nitrogen, the coverslip removed and the slide incubated in ethanol for 10 min at −20 °C. The brains were rehydrated for 10 min in PBS and mounted in DAPI Vectashield (Vector Laboratories).

For fluorescent *in situ* hybridization (FISH), mid-development pupae were dissected and fixed in 4% formaldehyde, transferred to 2XSSCT, and treated with RNase for 30 minutes. Samples were stepped into 50% formaldehyde/2XSSCT and prehybed for 4 hrs. 5 pmol of Dodeca satellite probe labeled with Cy3 was mixed in hybe solution (2XSSCT, 50% formaldehyde, and 10% dextran sulfate), added to samples, heat denatured at 91 °C for 2 min, and transferred to 37 °C overnight. Samples were washed 3X in 50% formaldehyde/2XSSCT at 37 °C, stepped into 2XSSCT, and briefly incubated with DAPI. Retinas were imaged using a Leica Sp8 (0.2 μm stacks), and Imaris was used to quantify puncta per cell. 3 confocal stacks from each genotype were processed using background subtraction thresholding, median filter smoothing (3 × 3 × 3), and the spot function to identify FISH puncta. Each spot was manually inspected to verify separate puncta, and nuclei were manually sorted into 1, 2 or ≥3 puncta per cell.

Adult brains were dissected in Sang Medium and fixed in a mix of acetic acid: methanol: water (11: 11: 2) for 30 seconds. Brain was transferred in 45% acetic acid for 2 min before being squash. Then, the slide was frozen in liquid nitrogen, the coverslip removed and the slide incubated in absolute ethanol for 10 min at −20 °C. The slides were air dry and stored at 4 °C. The slides were dehydrated at room temperature in 70%, 90% and absolute ethanol for 3 min prior to DNA denaturation in 70% formamide-2xSCC solution for 2 min at 70 °C. Then the slides were transferred to cold 70% Ethanol (−20 °C) and dehydrated at room temperature in 90% and absolute ethanol for 3 min. Hybridization was done over-night at 37 °C using 100 ng of pre-labelled Dodeca satellite probe in 50% formamide-1% dextran sulfate-2xSCC. Then, slides were washed 3 × 5 min in 50% formamide-2xSCC at 42 °C and 3 × 5 min in 0.1xSCC at 60 °C. Finally, the slides are washed in PBS, and mounted in DAPI Vectashield (Vector Laboratories, Burlingame, CA). Images were acquired using a Zeiss 710 confocal microscope.

### Cell Culture and mitotic spindle analysis

Stable inducible SH-S5Y5 cell lines expressing Tau 1N4R are described in ref. 36. Cells were maintained in DMEM medium with high glucose, sodium pyruvate (Life Technologies) and supplemented with 2 mM L-glutamine, 50 U/mL penicillin/streptomycin, 10% FBS, 5 μg/mL blasticidin and 100 μg / mL zeocin (Life Technologies). Medium was replenished every 3 days. Cell were plated on cover slip with fibronectin (Sigma) and Tau expression was induced for 96 hours with 1 μg/ml tetracycline (Sigma) prior to fixation in 4% paraformaldehyde for 30 min. Cells were permeabilized in PBST for 10 min. Next the slides were washed in PBS and blocked with 1% BSA in PBS for 45 min. Mouse anti-γ tubulin (Sigma, clone GTU-88) and Rabbit anti-phospho Histone H3 Ser 10 (Millipore) were used at 1:1000 in PBS and incubated for 1 h at RT. The slides were washed 3 × 5 min in PBS. Fluorescent secondary antibody (Invitrogen) were used at 1:1000 and incubated for 1 h at RT in PBS-1% BSA. Mouse anti-α tubulin-FITC conjugated (Sigma, clone DM1A) was used at 1:50 and incubated for 1 h at room temperature. Finally, the slides are washed in PBS, and mounted in DAPI Vectashield (Vector Laboratories, Burlingame, CA). Images were acquired using a Zeiss 780 confocal microscope.

### Orcein staining and CID detection

For Orcein staining, brains from 3^rd^ instar larvae were dissected in physiological saline solution (0.7% NaCl) and fixed in a mix of acetic acid: methanol: water (11: 11: 2) for 30 seconds. Then, the brains were transferred into a 2% aceto-orcein solution prior to be squash and frozen in liquid nitrogen.

For CID detection, whole mount adult brains carrying a CID-GFP transgene (16518797) were dissected in Sang medium. Following fixation in 4% paraformaldehyde for 30 min, brains were washed in PBST and blocked with 1% BSA in PBST for 45 min. Mouse anti-Elav antibody (DSHB, clone Elav-9F8A9) and Rabbit anti-GFP (Invitrogen) were used at 1:100 and 1:1000 respectively in PBST and incubated for 2 h at RT. The slides were washed 3 × 5 min in PBST. Fluorescent secondary antibody (Invitrogen) were used at 1:1000 and incubated for 1 h at RT in PBST. Finally, the slides are washed in PBS, and mounted in DAPI Vectashield (Vector Laboratories, Burlingame, CA). Images were acquired using a Zeiss 710 confocal microscope.

### Western Blot

10 fly heads were dissected and crushed into 30 μL NuPAGE^®^ LDS sample buffer + reducing agent (Life Technologies). Samples were centrifuged and the supernatant stored at −80 °C. Samples were loaded and separated onto a 4–12% acrylamid gel (NuPAGE^®^, Novex, Life Technologies), blotted on nitrocellulose membranes using Biorad Trans-Blot transfer system kit (Biorad) according to manufacturer’s technical recommendations. Membranes were blocked and probed with rabbit anti-Tau (Dako, 1:10000) and rabbit anti-Actin (Sigma, 1:2000). Membranes were incubated with a Horseradish Peroxidase-conjugated secondary antibody (Biorad), and revealed by chemiluminescence (ECL, Amersham) using a ChemiDoc™ XRS + System with Image Lab™ Software (Biorad).

### Live imaging of mitotic cells in third instar larval brains

Wild-type and Tau 2N4R expressing larval brains were dissected and process according to ref. 64 and observed using an inverted confocal microscope (Zeiss LSM710). The protein trap Jupiter-GFP was used to follow microtubule dynamics and Histone-RFP transgene to follow DNA movement^65^. The CNN-GFP transgene was used to follow centrosome movement (Megraw *et al*. 2002). Mitotic timing was calculated using Image J software (NIH) (“Time Stamper” plugin).

## Additional Information

**How to cite this article:** Malmanche, N. *et al*. Developmental Expression of 4-Repeat-Tau Induces Neuronal Aneuploidy in *Drosophila* Tauopathy Models. *Sci. Rep.* **7**, 40764; doi: 10.1038/srep40764 (2017).

**Publisher's note:** Springer Nature remains neutral with regard to jurisdictional claims in published maps and institutional affiliations.

## Supplementary Material

Supplementary Figures

Supplementary Movie 1

Supplementary Movie 2

Supplementary Movie 3

Supplementary Movie 4

Supplementary Movie 5

Supplementary Movie 6
