# Supplementary material for: Developmental Expression of 4-Repeat-Tau Induces Neuronal Aneuploidy in Drosophila Tauopathy Models
Source: Sci Rep. 2017 Jan 23;7:40764. doi: 10.1038/srep40764 (PMC5256094; doi:10.1038/srep40764)
Supplement: Supplementary Figures [file srep40764-s1.pdf]

Developmental Expression of 4-Repeat-Tau Induces Neuronal Aneuploidy in *Drosophila* Tauopathy Models

Nicolas Malmanche, Pierre Dourlen, Marc Gistelinck, Florie Demiautte, Nichole Link, Cloé Dupont, Lies Vanden Broeck, Elisabeth Werkmeister, Philippe Amouyel, Antonino Bongiovanni, Hélène Bauderlique, Dieder Moechars, Anne Royou, Hugo J. Bellen, Frank Lafont, Patrick Callaerts, Jean-Charles Lambert, Bart Dermaut

Supplementary Information

Supplementary figures:

Figure supplement 1: 3R-Tau isoform expression does not induce an increase in mitotic cells within the second mitotic wave (SMW) of the eye imaginal disc using the eye-specific or the panneuronal drivers. (A) Larval eye discs from wild-type, 4R-Tau and 3R-Tau were analyzed for M phase (using pH3 staining) and hTau using the *GMR-Gal4* driver (Scale bar = 50µm). (B) Quantification of pH3-positive mitotic cells SMW in wild-type (n=12), 4R-Tau (n=9) and 3R-Tau (n=12) using the *GMR-Gal4* driver (mean ± SD). (C) Larval eye discs from wild-type, 4R-Tau and 3R-Tau were analyzed for M phase (using pH3 staining) and hTau using the *Elav-Gal4* driver (Scale bar = 50µm). (D) Quantification of pH3-positive mitotic cells within the SMW in wild-type (n=7), 4R-Tau (n=7) and 3R-Tau (n=8) using the *Elav-Gal4* driver (mean ± SD).

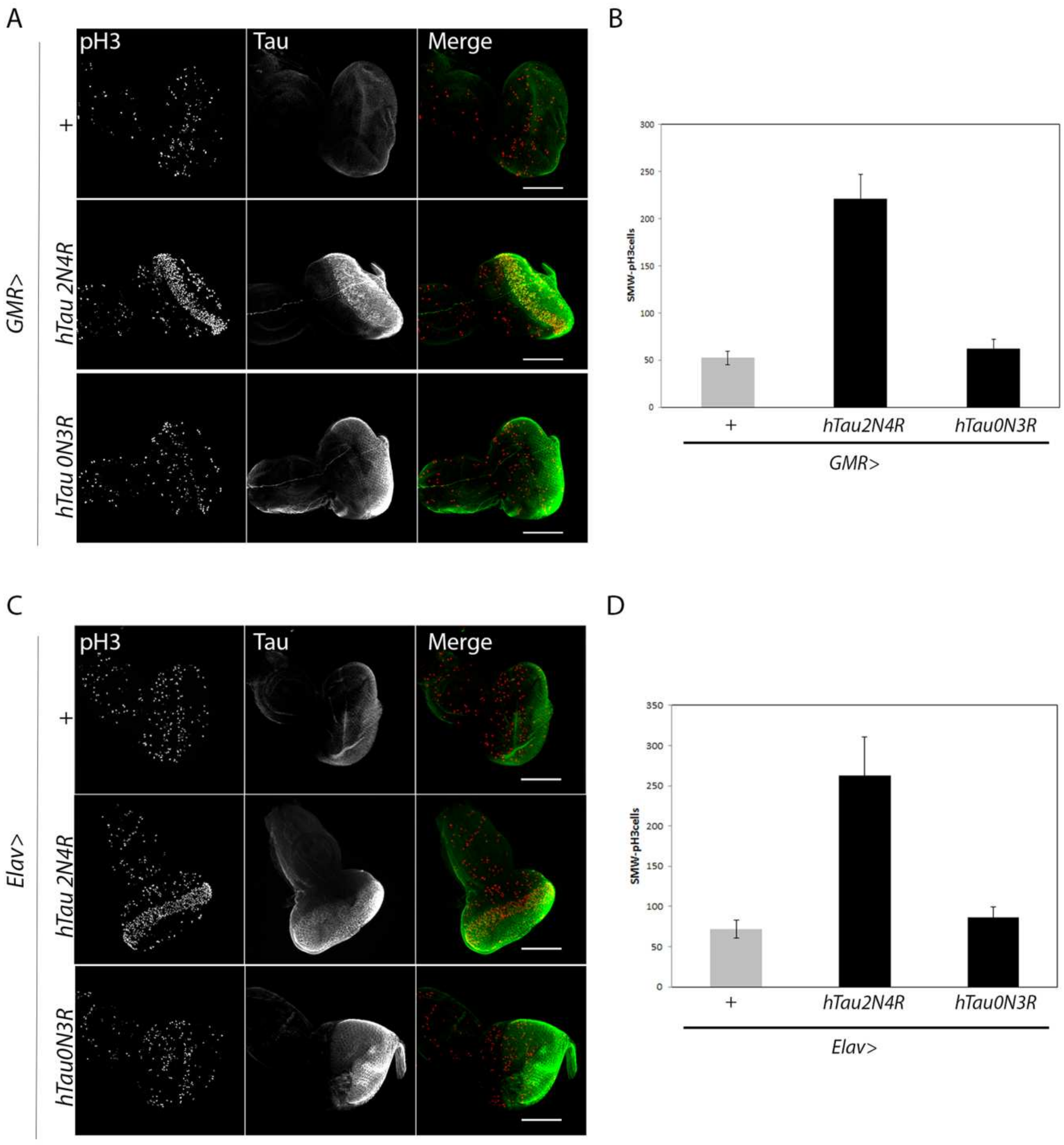
A
pH3
Tau
Merge
+
hTau 2N4R
hTau 0N3R
GMR>
B
SMW-pH3cells
300
250
200
150
100
50
0
+
hTau2N4R
hTau0N3R
GMR>
C
pH3
Tau
Merge
+
hTau 2N4R
hTau0N3R
Elav>
D
SMW-pH3cells
350
300
250
200
150
100
50
0
+
hTau2N4R
hTau0N3R
Elav>

Figure supplement 2: Panneuronal *Elav-Gal4* activity occurs prior to neuronal differentiation. (A) The *Elav-Gal4* driver induces GFP:CD8 localization within the neuroblast (asymmetric miranda staining). (B) The *Elav-Gal4* driver induces Tau expression at the same time as GFP:CD8.

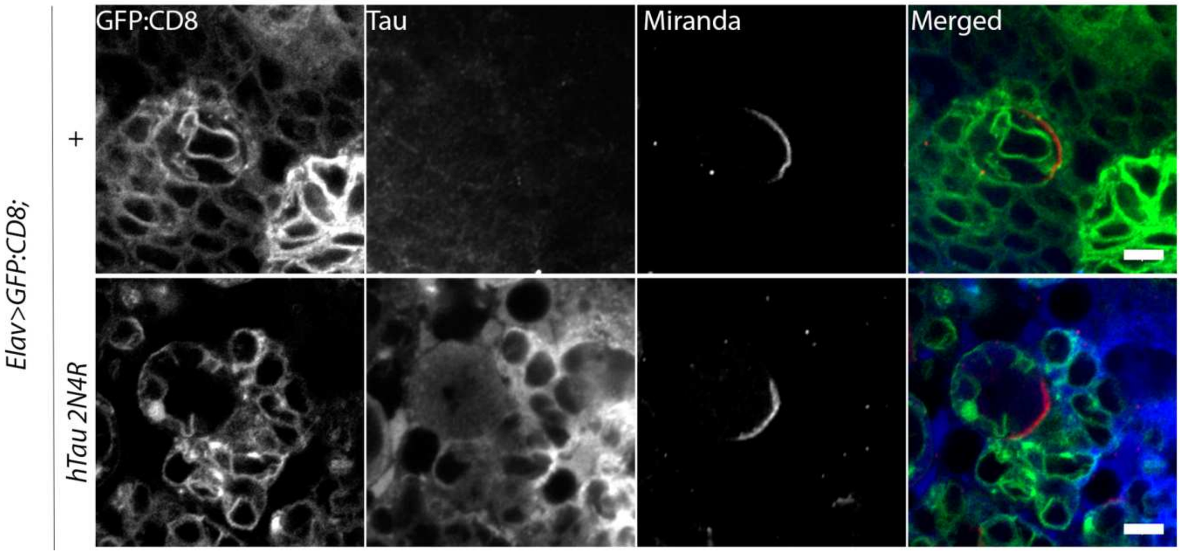

Figure supplement 3: 4R-Tau accumulation of mitotic cells in larval brains. (A) In control brains, at prometaphase/metaphase, cells have well-aligned chromosomes (arrows) whereas 4R-Tau-expressing cells exhibited hypercondensed and misaligned chromosome (arrowheads). Furthermore, the mitotic cells appear aneuploid. (B) Mitotic index in *Elav>, Elav>4R-Tau* and *Elav>3R-Tau* expressing third instar larval brains (n=4 for each genotypes, mean ± SD) indicates an accumulation of pH3-positive cells only in the presence of 4R-Tau isoforms.

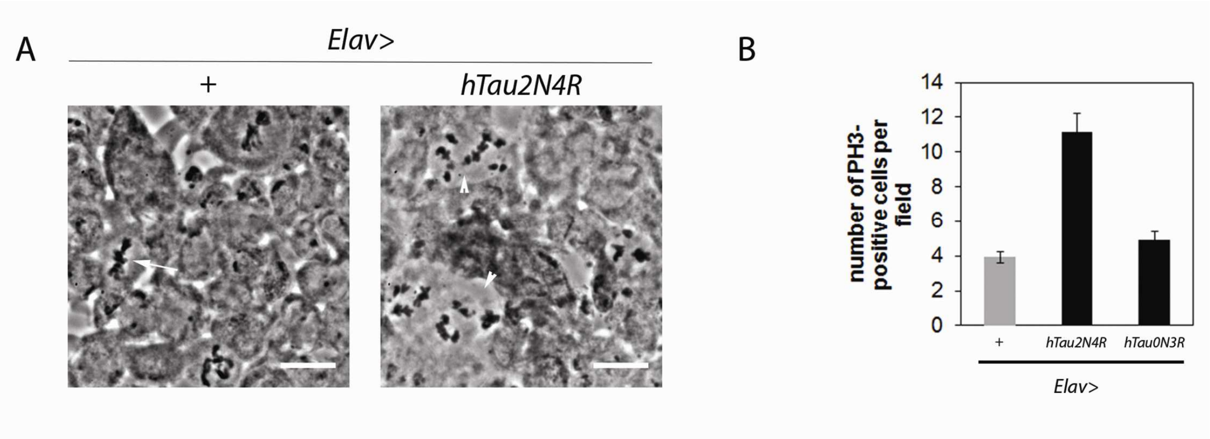

Figure supplement 4: Expression of 3R-Tau does not impact ploidy in pupal retinas. (A) Quantification of FISH foci in wild type and 3R-Tau expressing pupal retinas.

A

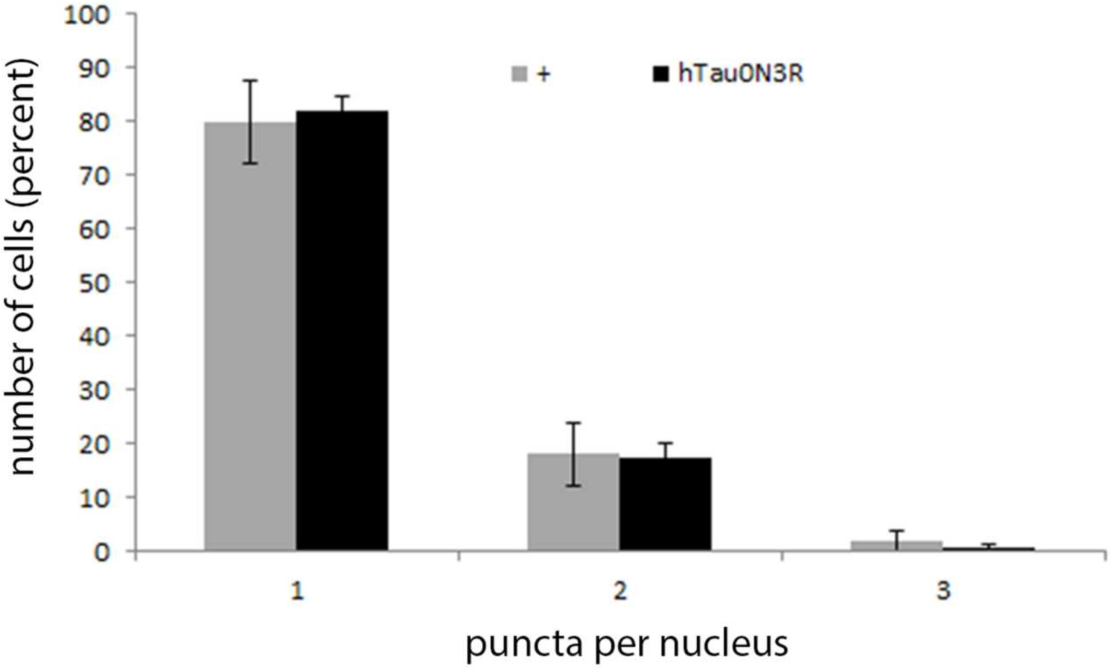

Figure supplement 5: Full-length versions of cropped gels/blots displayed in the main Figures. (A) Full-length western blot related to figure 1B and 2B. (B) Full-length western blot related to figure 3B and D. (C) Full-length western blot related to figure 7B.

Western blot corresponding at figure 1b and 2b and 2F

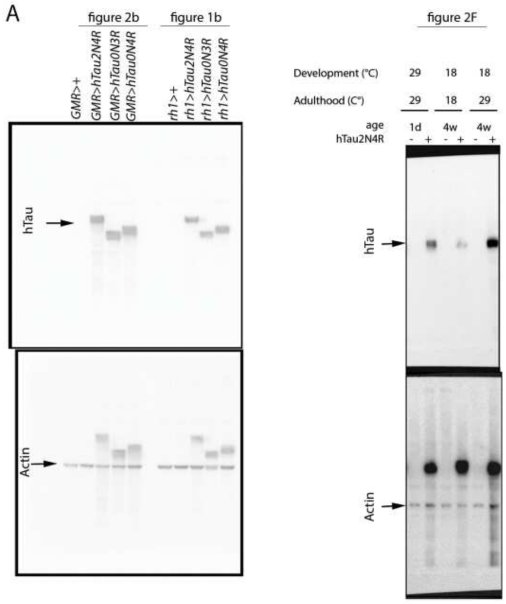

Western blot corresponding at figure 3B and D

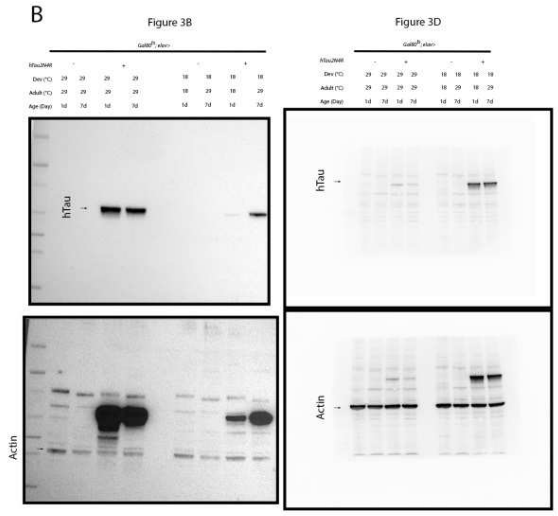

Western blot corresponding at figure 7

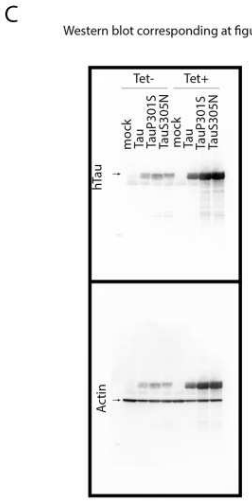

Supplementary movies – legends:

Movie S1: Mitosis in wild type *Jupiter:GFP; Histone:RFP* ganglion mother cell showing the formation of the bi-polar spindle and the segregation of two equal DNA masses (Scale bar = 5µm).

Movie S2: Mitosis in *Elav>hTau2N4R; Jupiter:GFP; Histone:RFP* ganglion mother cells showing a mitotic cell with a monopolar spindle and hypercondensed chromosome. The cell was already in mitosis at the beginning of the movie and we only captured mitotic exit after almost 2 hours. Furthermore, at anaphase, DNA segregation is asynchronous and unequal (Scale bar = 5µm).

Movie S3: Mitosis in *Elav>hTau2N4R; Jupiter:GFP; Histone:RFP* ganglion mother cells showing three cells undergoing mitosis, the first and second cells going through chromosome hypercondensation and spindle collapse and the third cell having a normal mitotic progression. The first cell (first arrow) has a long mitotic timing (30 min), the second cell divides in 20 min (second arrow) and the third cell divides in 17 min, with (arrowhead) within the same optical view (Scale bar = 5µm).

Movie S4: Mitosis in *Elav>hTau2N4R; Jupiter:GFP; Histone:RFP* ganglion mother cells showing a mitotic cell with a circular configuration and hypercondensed chromosome. The cell was already in mitosis at the beginning of the movie (Scale bar = 5µm).

Movie S5: Mitosis in *Elav>UAS-CNN:GFP; Histone:RFP* ganglion mother cell showing that each centrosome remains at a spindle pole after separation and during mitotic progression (Scale bar = 5µm).

Movie S6: Mitosis in *Elav>hTau2N4R; UAS-CNN:GFP; Histone:RFP* ganglion mother cell showing that centrosomes undergo cycle of separation and collapse prior to anaphase onset (Scale bar = 5µm).
